# Building Osteogenic Microenvironments With Strontium-Substituted Calcium Phosphate Ceramics

**DOI:** 10.3389/fbioe.2020.591467

**Published:** 2020-10-07

**Authors:** Ben Wan, Renxian Wang, Yuyang Sun, Jingjing Cao, Honggang Wang, Jianxun Guo, Dafu Chen

**Affiliations:** Laboratory of Bone Tissue Engineering, Beijing Laboratory of Biomedical Materials, Beijing Research Institute of Traumatology and Orthopaedics, Beijing Jishuitan Hospital, Beijing, China

**Keywords:** calcium phosphate ceramics, strontium substitution, microenvironment, bone regeneration, biomaterials

## Abstract

Bioceramics have experienced great development over the past 50 years. Modern bioceramics are designed to integrate bioactive ions within ceramic granules to trigger living tissue regeneration. Preclinical and clinical studies have shown that strontium is a safe and effective divalent metal ion for preventing osteoporosis, which has led to its incorporation in calcium phosphate-based ceramics. The local release of strontium ions during degradation results in moderate concentrations that trigger osteogenesis with few systemic side effects. Moreover, strontium has been proven to generate a favorable immune environment and promote early angiogenesis at the implantation site. Herein, the important aspects of strontium-enriched calcium phosphate bioceramics (Sr-CaPs), and how Sr-CaPs affect the osteogenic microenvironment, are described.

## Introduction

Bone is a metabolically active specialized connective tissue with the capacity for continuous resorption and reformation, but the reconstruction process requires extra support in large bone defect cases, for example, injury, tumor excision, and age-related diseases (Loi et al., 2016; Ginebra et al., 2018). Clinically, autografts still remain the most common strategy to support and stimulate bone growth. While biologically desirable, the supply of autografts from a patient’s own body cannot meet the amount required for large-sized bone defects (Wang and Yeung, 2017). Grafts from bone banks or other animal species can provide a vast range of grafting forms by virtue of their relatively easy availability (Wang and Yeung, 2017). Nevertheless, they are still subject to attenuated osteointegration and disease transmission.

Synthetic bioactive ceramics can overcome these limitations. They are obtained by chemical reagent deposition through a controlled process that allows their properties to be adapted to the specific requirements of different clinical situations. Calcium phosphate ceramics (CaPs) are basic bone repair materials with excellent osteoinductive and osteoconductive features; examples include hydroxyapatite (HAP), β-tricalcium phosphate (β-TCP), calcium polyphosphate (CPP), and biphasic calcium phosphate (BCP). They have been widely used in clinical applications in the form of implant surface coatings, the composition of cements, and scaffolds (Jeong et al., 2019). Moreover, CaPs encapsulated with pharmacologics and biologics in scaffolds have shown multiple abilities, such as enhanced osteointegration, angiogenesis, and antibacterial properties (Bose and Tarafder, 2012; Marques et al., 2016). However, these biologically active molecules are only surface-loaded and show explosive release effects with little long-term benefit (Wu et al., 2014). This raises some concerns about their safety. For example, recombinant human bone morphogenetic protein-2 (rhBMP-2) is the first commercial synthetic osteoinductive molecule, and the combined use of rhBMP-2 and calcium phosphate ceramics has achieved many clinical successes. But it is worth noting that serious side effects related to ectopic or unwanted bone formation in certain situations have made the FDA increasingly resistant to approving the use of such materials (Vavken et al., 2016).

An alternative long-lasting and potentially safer strategy is the incorporation of trace metallic elements into CaPs. The mineral composition of our bone itself is not a homogenous and bioinert calcium phosphate-based material (Von Euw et al., 2019). Bone incorporates and releases various trace elements (such as Mg^2+^, Zn^2+^, and Sr^2+^) into the microenvironment during bone metabolism (Bose et al., 2013). Previous studies have confirmed that CaPs modified with these bivalent trace metallic ions can lead to controlled degradation, increase the mechanical strength of the materials, and enhance bioactive properties. But among these bioactive ions, strontium (Sr) has gained great attention since strontium ranelate (SrRan) has been approved as an anti-osteoporotic drug for post-menopausal osteoporosis since it increases bone strength (Neves et al., 2017; Marx et al., 2020). Recent findings suggest that oral administration of SrRan may have no anabolic action on bone formation in humans, and even inhibits osteogenic differentiation under the *in vitro* experiment conditions (Wornham et al., 2014; Marx et al., 2020). However, the comprehensively described SrCaPs do exert positively influence on new bone formation and accelerate the healing process (Neves et al., 2017). These discrepancies may be partially due to the implant microenvironments where biomaterials interact with various cells. The local delivery of Sr ions from SrCaPs implanted in bone changes local microenvironment, which is involved in several biological processes such as osteogenesis, angiogenesis, osteoimmunomodulation.

Consequently, in this review, we present recent developments in strontium-substituted calcium phosphate ceramics (SrCaPs) and offer insights into how Sr ions released from implants influence the immune response, angiogenesis, and osteoblastic differentiation of bone marrow stromal cells (bMSCs).

## Sr-CaPs: Current State of the Art

In the 1960s, surgeons valued the inertness of surgical materials in the human tissue environment, and first-generation bioceramics (alumina, zirconia, diverse forms of carbon) were developed as bone substitutes, mainly for femoral head fabrication (Smith, 1963). However, they elicited a foreign body reaction, forming fibrous capsules that isolated them from the body. In the 1980s, Larry Hench invented a bioactive glass with the ability to bond to living tissue, and Heughebaert reported that CaPs could induce bone formation (Hench and Wilson, 1984; Heughebaert et al., 1988). Since then, osteoinductive and tissue-inducing materials have become the mainstream of biomaterial science and engineering (Hench and Polak, 2002). CaPs are the most studied and implanted bioactive ceramics in the clinic because of their cost-effective preparation from chemical regents or natural resources. Various calcium phosphate compounds are in different crystalline phases and often classified on the basis of the Ca/P ratio. Of great interest is the subgroup of apatite compounds, which exhibit similarities to vertebrate hard tissues. Indeed, both bone and teeth are well-crystallized apatites (hydroxyapatite), and this structure can easily accommodate many cations and anions for substitution, thereby achieving adaptive biophysical functions (Drouet et al., 2017).

Strontium (Sr) is naturally deposited in the mineral phase of bones via consuming a normal diet, and it replaces approximately 0.035% of Ca content (International Programme on Chemical and Inter-Organization Programme for the Sound Management, 2010). The majority of *in vitro* experiments support a dual-acting mechanism in which Sr stimulates bone formation and hinders bone resorption (Saidak and Marie, 2012). The exact mechanism of the role of Sr in bone remains unclear, but it has been proposed that Sr acts on similar cellular targets as Ca^2+^ by activating the calcium sensing receptor (CaSR), thus interacting with Ca-driven signaling pathways related to bone metabolism regulation (Saidak and Marie, 2012). Furthermore, small animal trials and clinical trials have produced overwhelming evidence that Sr benefits bone remodeling and increases bone-mineral density, especially in the treatment of osteoporosis (Meunier et al., 2004; Reginster et al., 2012; Marx et al., 2020). Taking these beneficial effects into consideration, it is likely that local Sr ion delivery will enhance osteoinduction and osseointegration at the bone-implant interface, contributing to a faster healing microenvironment.

Recently, Sr has been widely incorporated into calcium phosphate-based materials for biomedical applications. Krishnan reported using Sr doped hydroxyapatite (SrHAP) particles to repair dental enamel with the potential to treat white spot lesions and incipient carious lesions (Krishnan et al., 2016). It is also an effective additive for toothpaste in the prevention of cariogenesis (Surdacka et al., 2007). These particles cannot only be used as a raw material, but also associated with other biomaterial compounds to facilitate new functions. Sr-CaPs are fabricated as coatings on the surfaces of metallic implants in order to accelerate bone healing at early implantation times (Li et al., 2010; Arcos and Vallet-Regí, 2020). Titanium and titanium-based alloys are commonly used for load-bearing applications (such as total joint replacement and fracture fixation elements), but carry a risk of loosening, especially when implanted in osteoporotic bones. Tao et al. (2016a) conducted an *in vivo* study on rats with ovariectomy-induced osteoporosis and reported that a strontium-containing HAP coating on shaped titanium implants was better than an Sr-free HAP coating in terms of new bone formation and push-out force. Furthermore, they conducted an comparative study that found that under the same the molar ratio [Zn, Mg, Sr/(Zn, Mg, Sr+Ca) = 10%], the SrHAP coating exhibited better osseointegration than zinc- and magnesium-substituted HAP coatings (Tao et al., 2016b).

SrCaP cements (SrCPC) consist of a combination of a precursor powder and liquid phase, allowing it to fill and heal bone defects during minimally invasive procedures. Kuang et al. (2015) prepared pre-set SrCPC for a metaphyseal defect in Sprague-Dawley rats and found early endochondral ossification at 2 weeks post-operation. Thormann et al. (2013) further demonstrated that Sr was deposited at newly formed tissues around the implanted SrCPC as visualized by Time-of-flight secondary ion mass spectrometry (ToF-SIMS) imaging and found a significant increase of mineralized extracellular matrix in the SrCPC group compared to the Sr-free calcium phosphate cement (CPC) and empty defect groups. Sr-CaP scaffolds can be further modified with drugs, especially antibiotics and growth factors. The chemical composition of SrHAP provides strong surface adsorption capacity and increases surface area, allowing drug release for a longer period of time (Xu et al., 2018). Lin et al. (2013) reported that SrHAP microspheres with 3D hierarchically mesoporous structures not only promoted an osteogenic response, but exhibited sustained vancomycin release. Yan et al. (2018) developed an Sr-nHAP/SF-Hep-BMP-2 scaffold system with controlled BMP-2 release that supported critical size calvarial defect healing in Sprague-Dawley rats. Tao et al. (2018) revealed synergistic bioactive properties of BMP-2 and Sr released from CPC composites, which achieved rapid bone healing in osseous defects in an ovariectomized rat model. These new applications for strontium-containing calcium phosphate extends their biomedical potential.

## Favorable Post-Implantation Environments for Sr-CaPs

**FIGURE 1:**
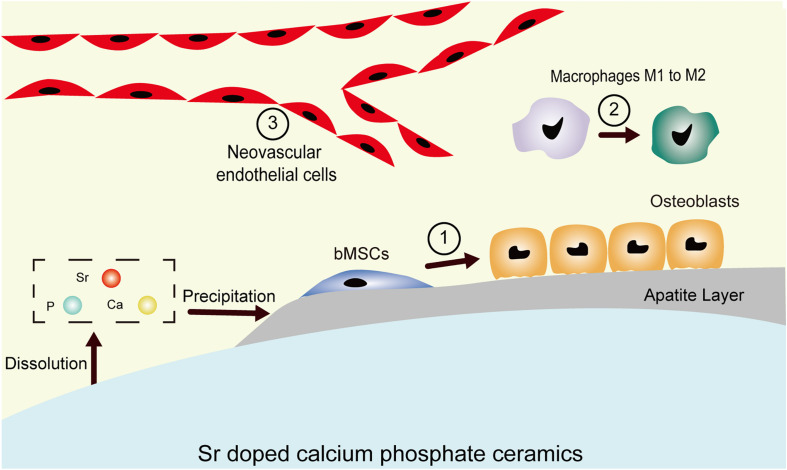
Schematic demonstrating the microenvironment of the host bone-implant interface. The degradation-precipitation reactions of bioactive Sr-CaPs modulate local ion concentrations and influence peripheral physiological processes, including ① hMSC osteogenic differentiation, ② immune responses such as macrophage polarization to M2, and ③ revascularization processes.

Sr is chemically and physically close to calcium but has a larger ionic radius (112 vs. 99 pm). The partial substitution of Ca by Sr results in higher solubility when compared with Sr-free CaPs owning to the enlargement of the unit cell (Zhu et al., 2018). The surfaces of SrCaPs are reactive and biodegradable, and bone-like apatite forms on them through continuous dissolution and precipitation. The newly formed apatite creates a strong bond between the living tissues and implants. However, it degrades over time when in contact with bodily fluids and it elevates the local concentrations of Sr^2+^, Ca^2+^, and PO_4_^3–^ ions. These released ions affect important components of the bone microenvironment, including the mineral deposited layer phase, the cellular phase (osteoblasts, macrophages, and vascular endothelial cells), and the soluble factor phase (growth factors and/or cytokines) and provide an extracellular osteogenic signaling network. The incorporation of an appropriate proportion of Sr into CaPs allows them to build an osteogenic microenvironment via modulating the inflammatory response, stimulating osteogenic differentiation of bone marrow mesenchymal stem cells, and promoting early angiogenesis (Figure 1).

## Effects on Osteogenesis

SrCaPs is a promising biomaterial to help bone reconstruction. Mounting evidence shows that Sr replacement of Ca in CaPs leads to increased solubility due to the expansion of the crystal structure, and the high local Sr^2+^, Ca^2+^, and PO_4_^3–^ ionic concentrations are believed to be of great importance for osteoinduction and osteoconduction (Bose et al., 2013; Neves et al., 2017). Current research shows that both Sr and Ca mediate key cellular functions in osteogenesis-related cells via Ca-sensing receptor (CaSR)-dependent mechanisms (Marx et al., 2020). In the extracellular microenvironment, a high Ca concentration is a potent chemical signal for bMSCs osteogenic differentiation (Dvorak and Riccardi, 2004; Barradas et al., 2012). A lack of calcium significantly retards cell growth and differentiation (Dvorak et al., 2004; Barradas et al., 2012). Interestingly, CaPs with Sr modification tend to have decreased Ca concentrations around the material due to induced apatite precipitation, though lower concentrations of Ca do not impair the proliferation or osteogenic differentiation of bMSCs (Schumacher et al., 2013; Kruppke et al., 2019b). In an *in vitro* study, Kruppke et al. (2019a) confirmed that Sr may compensate for the adverse effects of lowered Ca ion concentrations to a certain degree and potentially stimulate bone regeneration. They investigated the effect of varied Ca and Sr ion concentrations, respectively, on proliferation and differentiation of bMSCs and concluded that under low calcium concentrations culture conditions (0.3–0.4 mM due to the addition of fetal calf serum), Sr at 0.4–0.9 mM promoted differentiation of bMSCs while 0.9–1.8 mM stimulated the highest proliferation. And in a second study exploring the relationship between Sr and Ca on bone formation, they conducted *in vitro* and *in vivo* experiments to evaluate the osteogenesis effects of Sr together with two concentration gradients of Ca (normal calcium of 1.8 mM or high calcium of 9 mM) and concluded that Sr (1 mM) enhanced mineralization and ALP expression of MC3T3-E1 pre-osteoblast cells under a high dose of calcium (9 mM) (Xie et al., 2018). This may explain the results by Wornham et al. (2014) in which concentrations of Sr ranging from 10 μM to 1 mM inhibit the mineralization ability of primary rat osteoblasts under 1.8 mM Ca present in the culture system. These experiments illustrate the importance of the implant microenvironment because bone has higher Ca levels than plasma. It also suggests that both Sr and Ca from ion-leaching SrCaPs may interact to promote bone regeneration at the bone-implant interface. In addition, the phosphate ion is a basic component in the human body and accumulates in bone minerals in the form of calcium phosphates along with calcium ions (Khoshniat et al., 2011). When phosphate ions are not present in high quantities, the generation of new bone will be blocked due to insufficient formation of hydroxyapatite (Penido and Alon, 2012).

Sr-rich CaPs consistently outperform Sr-free CaPs in *in vitro* and *in vivo* studies (Neves et al., 2017; Marx et al., 2020). In the absence of doping with other osteogenic ions, the local release of Sr is sufficient to promote bone formation, but it is difficult to ignore the effects that surface morphology, porosity, hydrophilicity, dissolution process, and local pH changes may have on osteogenesis regulation at the implantation site.

## Effects on Angiogenesis

Increased angiogenesis can relieve the symptoms of ischemia and facilitate the transport nutrients, which is essential for bone repair/regeneration. Sr-CaPs have been found to increase neovascularization in many convincing experiments (Liu et al., 2011; Gu et al., 2013; Wang et al., 2014). Endothelial cells are the major seed cells that drive angiogenesis. Chen et al. (2008) observed that degradation products of Sr doped calcium polyphosphate (SCPPs) promote the proliferation, migration, and tube-like structure formation of endothelial cells (ECV304). It has been reported that *in situ* production of angiogenic growth factors, such as vascular endothelial growth factor (VEGF) and basic fibroblast growth factor (bFGF), can promote more cell infiltration, extracellular matrix synthesis, and faster angiogenesis (Saberianpour et al., 2018). Liu et al. (2011) directly seeded osteoblasts (ROS17/2.8) on SCPP scaffolds with various amounts of Sr and found increased secretion of VEGF and bFGF in the culture medium. Wang et al. (2014) observed the stimulating effects of SCPP on protein secretion and mRNA expression of VEGF, bFGF, and MMP-2 from endothelial cells *in vitro*. Gu et al. (2014) co-cultured umbilical vein endothelial cells and osteoblasts on SCPP scaffolds *in vitro* and demonstrated higher VEGF and bFGF protein production compared with CPP and HAP. Furthermore, Ye et al. (2019) fabricated Sr-doped calcium phosphate/polycaprolactone/chitosan (Sr-CaP/PCL/CS) nanohybrid fibrous membranes and demonstrated high VEGF secretion from bMSCs. In the future, more experiments are needed to test the ability of SrCaPs to promote blood vessel formation *in vivo*, and more attention should be paid to the impact of the immune response.

## Effects on Osteoimmunomodulation

Every implantable material must be biocompatible, meaning that only a limited inflammatory response occurs in the host body. Huang et al. (2020) reported the incorporation of fish-derived nano CaPs (Ca/P molar ratio was 2.35) into gelatin methacrylate generated a suitable immune microenvironment by inducing the secretion of cytokines and promoting macrophage phenotype conversion. The controllable inflammation may promote the osteogenic differentiation and angiogenesis (Zhao et al., 2018; Huang et al., 2020). The local release of Sr from implants to the adjacent host bone can reduce unfavorable inflammatory responses (Renaudin et al., 2008), and Sr has been proven to be an anti-inflammatory agent (Wang et al., 2020). Buache et al. (2012) first verified that Sr-BCP can decrease the production of pro-inflammatory cytokines (TNF-α and IL-6) and the chemokine interleukin 8 in human monocytes. Braux et al. (2016) demonstrated the downregulating effect of Sr-BCP on inflammatory mediator production (MCP-1 and Gro-α) by human primary osteoblasts. Sr has also been shown to suppress the expression of proinflammatory cytokines (IL-6 and TNF-α) in periodontal ligament cells and macrophages (Römer et al., 2012; Gu et al., 2014). Among various immune-related cells, macrophages play a significant role in regulating bone healing after trauma or the implantation of biomaterials (Alexander et al., 2011; Batoon et al., 2019). At the early time of bone repair, resident or infiltrating monocyte-derived macrophages mostly present the pro-inflammatory M1 phenotype, and a timely switch from M1 to M2 (anti-inflammatory phenotype) leads to osteogenic cytokine release, including that of IL-10, TGFβ, and VEGF. Interestingly, prolonged M1 polarization leads to an increased release of fiber-enhancing cytokines from M2 macrophages, which leads to the formation of fibrous capsules (Chen et al., 2016). Previous evidence suggests that Sr-integrated implants elicit more pro-regenerative M2 macrophages; examples include Sr-doped calcium polyphosphate particles, Sr-substituted bioactive glass, and Ca- and Sr-modified titanium implant surfaces (Gu et al., 2014; Lee et al., 2016; Zhao et al., 2018). The incorporation of Sr into CaPs endows traditional CaPs with osteoimmunomodulation properties and positively regulates cytokine production and immune cell functions for better bone remodeling.

## Controversy Over the Effective Strontium Concentration

Local Sr release is a critical property of SrCaPs, but the optimal concentration remains to be determined. Evidence from *in vitro* studies shows that Sr promotes osteogenic differentiation in a dose-dependent manner, and different cell lineages respond differently to Sr at similar concentrations (Table 1). Peng et al. (2009) showed that 1 mM can promote osteogenic differentiation of C3H10T1/2 or mice primary bMSCs, while Aimaiti et al. (2017) reported that 1 mM induces apoptosis and inhibits osteogenic differentiation in human primary adipose-derived stem cells. Moreover, inhibition at this concentration also occurs for primary rat osteoblastic cells and MC3T3-E1 cells (Wornham et al., 2014; Xie et al., 2018). However, the maximum circulating concentrations of Sr in the serum of patients treated with strontium ranelate (2 g per day for 3 years) is about 0.1 mM (Meunier et al., 2004). Although several *in vitro* studies have shown that Sr concentration above 1 mM can stimulate osteogenesis, it is preferable to take clinical trial results into consideration, and the effective range (below 500 mM) is recommended here (Bonnelye et al., 2008; Peng et al., 2009; Guo et al., 2016). To the best of our knowledge, no *in vivo* study on the local administration of Sr with the use of modified CaPs has reported adverse effects on bone formation and osseointegration. The reasons may be that, first, the implantation microenvironment in the body is dynamically changing, and a high dose of Sr does not last for a long time. Second, the degradation product is not only Sr, but also Ca, which matters because of the equilibrium effect of Ca to Sr (Xie et al., 2018). Third, Sr positively modulates the microenvironment via regulating immune cells and endothelial cells in the process of bone formation.

**TABLE 1:** The osteogenic differentiation function of various Sr concentrations.

Cell lines	Strontium salt	Sr concentration	Function	References
C3H10T1/2 and mice primary bone marrow mesenchymal stem cells	Strontium chloride	1.0 mM, 3.0 mM	Promote osteogenic differentiation	Peng et al., 2009
Ovariectomy bone marrow mesenchymal stem cells	Strontium ranelate	0.25–0.5 mM	Promote osteogenic differentiation	Guo et al., 2016
Primary fetal mouse calvaria cells	Strontium ranelate	0.1–1 mM	Promote osteogenic differentiation	Bonnelye et al., 2008
Human bone marrow mesenchymal stem cells	Strontium ranelate	2.4–240 μM	Promote osteogenic differentiation	Sila-Asna et al., 2007
Human adipose-derived stem cells	Strontium ranelate	25–500 μM	Promote osteogenic differentiation	Aimaiti et al., 2017
	Strontium ranelate	1–3 mM	Apoptosis and inhibit osteogenic differentiation	
MC3T3-E1 pre-osteoblast cells	Strontium chloride	1 mM	Inhibit osteogenic differentiation	Xie et al., 2018
Rat bone marrow mesenchymal stem cells	Strontium ranelate	0.1 mM, 1 mM	Inhibit proliferation and promote osteogenic differentiation	Li et al., 2012
Primary rat osteoblastic cells	Strontium ranelate; strontium chloride	0.1–1 mM	Inhibit mineral deposition	Wornham et al., 2014

## Conclusion

**FIGURE 2:**
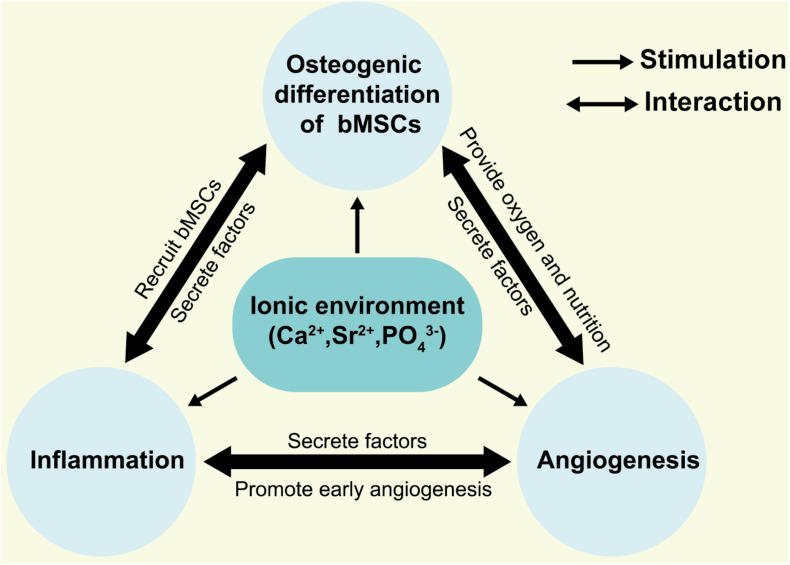
Hypothesis for osteogenic microenvironments of SrCaPs. The local ionic environment not only stimulates the osteoblastic differentiation of bMSCs but interacts with the inflammatory cells and vascular endothelial cells.

In summary, integrating Sr ions into CaPs offers an alternative to biologics in the design of bioactive materials. It is of low cost, has a longer shelf life, and presents low systemic risk compared to growth factors. These added benefits make Sr as therapeutic agent attractive in tissue engineering and regenerative medicine applications (Jiménez et al., 2019; Prabha et al., 2019; Mao et al., 2020). However, the mechanism of osteoinduction of SrCaPs remains complicated because inflammation and angiogenesis accompany the entire process of bone healing. In light of the previous studies, we propose a hypothesis for the osteoinduction mechanism of SrCaPs: SrCaPs build the osteoinductive microenvironment of the host bone-implant interface (Figure 2). Functional ion release and apatite layer formation on the surfaces of SrCaP ceramics allow them to interact with cells and extracellular matrices in the host system, providing bioactive bonding to bone. Specifically, a higher local concentration of Sr stimulates the osteoblastic differentiation of MSCs (1 in Figure 1), induces macrophage polarization toward a pro-regenerative M2 phenotype (2 in Figure 1), and contributes to angiogenesis (3 in Figure 1). Collectively, understanding the biological microenvironment of implant-to-tissue interactions at the bone site is important for the development of high-performance bioceramics.

## Author Contributions

BW was the first author and drafted the manuscript. DC and RW designed the work. YS, JG, JC, and HW summarized related publications. All authors approved the final version to be published.

## Conflict of Interest

The authors declare that the research was conducted in the absence of any commercial or financial relationships that could be construed as a potential conflict of interest.

## References

[B1] Aimaiti A. Maimaitiyiming A. Boyong X. Aji K. Li C. Cui L. (2017). Low-dose strontium stimulates osteogenesis but high-dose doses cause apoptosis in human adipose-derived stem cells via regulation of the ERK1/2 signaling pathway. *Stem Cell Res. Ther.* 8:282. 10.1186/s13287-017-0726-8 PMC5735894 29254499

[B2] Alexander K. A. Chang M. K. Maylin E. R. Kohler T. Müller R. Wu A. C. (2011). Osteal macrophages promote in vivo intramembranous bone healing in a mouse tibial injury model. *J. Bone Miner. Res.* 26 1517–1532. 10.1002/jbmr.354 21305607

[B3] Arcos D. Vallet-Regí M. (2020). Substituted hydroxyapatite coatings of bone implants. *J. Mater. Chem. B* 8 1781–1800. 10.1039/c9tb02710f 32065184 PMC7116284

[B4] Barradas A. M. C. Fernandes H. A. M. Groen N. Chai Y. C. Schrooten J. Van De Peppel J. (2012). A calcium-induced signaling cascade leading to osteogenic differentiation of human bone marrow-derived mesenchymal stromal cells. *Biomaterials* 33 3205–3215. 10.1016/j.biomaterials.2012.01.020 22285104

[B5] Batoon L. Millard S. M. Wullschleger M. E. Preda C. Wu A. C.-K. Kaur S. (2019). CD169+ macrophages are critical for osteoblast maintenance and promote intramembranous and endochondral ossification during bone repair. *Biomaterials* 196 51–66. 10.1016/j.biomaterials.2017.10.033 29107337

[B6] Bonnelye E. Chabadel A. Saltel F. Jurdic P. (2008). Dual effect of strontium ranelate: stimulation of osteoblast differentiation and inhibition of osteoclast formation and resorption in vitro. *Bone* 42 129–138. 10.1016/j.bone.2007.08.043 17945546

[B7] Bose S. Fielding G. Tarafder S. Bandyopadhyay A. (2013). Understanding of dopant-induced osteogenesis and angiogenesis in calcium phosphate ceramics. *Trends Biotechnol.* 31 594–605. 10.1016/j.tibtech.2013.06.005 24012308 PMC3825404

[B8] Bose S. Tarafder S. (2012). Calcium phosphate ceramic systems in growth factor and drug delivery for bone tissue engineering: a review. *Acta Biomater.* 8 1401–1421. 10.1016/j.actbio.2011.11.017 22127225 PMC3418064

[B9] Braux J. Velard F. Guillaume C. Jourdain M.-L. Gangloff S. C. Jallot E. (2016). Strontium-substituted bioceramics particles: a new way to modulate MCP-1 and Gro-α production by human primary osteoblastic cells. *Materials* 9:985. 10.3390/ma9120985 28774105 PMC5456992

[B10] Buache E. Velard F. Bauden E. Guillaume C. Jallot E. Nedelec J. M. (2012). Effect of strontium-substituted biphasic calcium phosphate on inflammatory mediators production by human monocytes. *Acta Biomater.* 8 3113–3119. 10.1016/j.actbio.2012.04.045 22579711

[B11] Chen Y. W. Feng T. Shi G. Q. Ding Y. L. Yu X. X. Zhang X. H. (2008). Interaction of endothelial cells with biodegradable strontium-doped calcium polyphosphate for bone tissue engineering. *Appl. Surface Sci.* 255 331–335. 10.1016/j.apsusc.2008.06.154

[B12] Chen Z. Klein T. Murray R. Z. Crawford R. Chang J. Wu C. (2016). Osteoimmunomodulation for the development of advanced bone biomaterials. *Mater. Today* 19 304–321. 10.1016/j.mattod.2015.11.004

[B13] Drouet C. Leriche A. Hampshire S. Kashani M. Stamboulis A. Iafisco M. (2017). “2 - types of ceramics: material class,” in *Advances in Ceramic Biomaterials*, eds Palmero P. Cambier F. De Barra E. (Cambridge, MA: Woodhead Publishing), 21–82.

[B14] Dvorak M. M. Riccardi D. (2004). Ca2+ as an extracellular signal in bone. *Cell Calcium* 35 249–255. 10.1016/j.ceca.2003.10.014 15200148

[B15] Dvorak M. M. Siddiqua A. Ward D. T. Carter D. H. Dallas S. L. Nemeth E. F. (2004). Physiological changes in extracellular calcium concentration directly control osteoblast function in the absence of calciotropic hormones. *Proc. Natl. Acad. Sci. U.S.A.* 101 5140–5145. 10.1073/pnas.0306141101 15051872 PMC387387

[B16] Ginebra M.-P. Espanol M. Maazouz Y. Bergez V. Pastorino D. (2018). Bioceramics and bone healing. *EFORT Open Rev.* 3 173–183. 10.1302/2058-5241.3.170056 29951254 PMC5994622

[B17] Gu Z. Xie H. Huang C. Peng H. Tan H. Li L. (2014). Effects of strontium-doped calcium polyphosphate on angiogenic growth factors expression of co-culturing system in vitro and of host cell in vivo. *RSC Adv.* 4 2783–2792. 10.1039/c3ra44323j

[B18] Gu Z. Zhang X. Li L. Wang Q. Yu X. Feng T. (2013). Acceleration of segmental bone regeneration in a rabbit model by strontium-doped calcium polyphosphate scaffold through stimulating VEGF and bFGF secretion from osteoblasts. *Mater. Sci. Eng. C* 33 274–281. 10.1016/j.msec.2012.08.040 25428072

[B19] Guo X. Wei S. Lu M. Shao Z. Lu J. Xia L. (2016). Dose-dependent effects of strontium ranelate on ovariectomy rat bone marrow mesenchymal stem cells and human umbilical vein endothelial cells. *Int. J. Biol. Sci.* 12 1511–1522. 10.7150/ijbs.16499 27994515 PMC5166492

[B20] Hench L. L. Polak J. M. (2002). Third-generation biomedical materials. *Science* 295 1014–1017. 10.1126/science.1067404 11834817

[B21] Hench L. L. Wilson J. (1984). Surface-active biomaterials. *Science* 226 630–636. 10.1126/science.6093253 6093253

[B22] Heughebaert M. Legeros R. Z. Gineste M. Guilhem A. Bonel G. (1988). Physicochemical characterization of deposits associated with HA ceramics implanted in nonosseous sites. *J. Biomed. Mater. Res.* 22 257–268. 10.1002/jbm.820221406 3235463

[B23] Huang L. Zhang J. Hu J. Zhao T. Gu Z. (2020). Biomimetic gelatin methacrylate/nano fish bone hybrid hydrogel for bone regeneration via osteoimmunomodulation. *ACS Biomater. Sci. Eng.* 6 3270–3274. 10.1021/acsbiomaterials.0c00443 33463163

[B24] International Programme on Chemical Inter-Organization Programme for the Sound Management (2010). *Strontium and Strontium Compounds.* Geneva: World Health Organization.

[B25] Jeong J. Kim J. H. Shim J. H. Hwang N. S. Heo C. Y. (2019). Bioactive calcium phosphate materials and applications in bone regeneration. *Biomater. Res.* 23:4. 10.1186/s40824-018-0149-3 PMC6332599 30675377

[B26] Jiménez M. Abradelo C. San Román J. Rojo L. (2019). Bibliographic review on the state of the art of strontium and zinc based regenerative therapies. Recent developments and clinical applications. *J. Mater. Chem. B* 7 1974–1985. 10.1039/c8tb02738b 32254801

[B27] Khoshniat S. Bourgine A. Julien M. Weiss P. Guicheux J. Beck L. (2011). The emergence of phosphate as a specific signaling molecule in bone and other cell types in mammals. *Cell. Mol. Life Sci.* 68 205–218. 10.1007/s00018-010-0527-z 20848155 PMC11114507

[B28] Krishnan V. Bhatia A. Varma H. (2016). Development, characterization and comparison of two strontium doped nano hydroxyapatite molecules for enamel repair/regeneration. *Dental Mater.* 32 646–659. 10.1016/j.dental.2016.02.002 26922626

[B29] Kruppke B. Heinemann C. Wagner A.-S. Farack J. Wenisch S. Wiesmann H.-P. (2019a). Strontium ions promote in vitro human bone marrow stromal cell proliferation and differentiation in calcium-lacking media. *Dev., Growth Diff.* 61 166–175. 10.1111/dgd.12588 30585307

[B30] Kruppke B. Wagner A.-S. Rohnke M. Heinemann C. Kreschel C. Gebert A. (2019b). Biomaterial based treatment of osteoclastic/osteoblastic cell imbalance – Gelatin-modified calcium/strontium phosphates. *Mater. Sci. Eng. C* 104:109933. 10.1016/j.msec.2019.109933 31499966

[B31] Kuang G.-M. Yau W. P. Wu J. Yeung K. W. K. Pan H. Lam W. M. (2015). Strontium exerts dual effects on calcium phosphate cement: accelerating the degradation and enhancing the osteoconductivity both in vitro and in vivo. *J. Biomed. Mater. Res. A* 103 1613–1621. 10.1002/jbm.a.35298 25087971

[B32] Lee C.-H. Kim Y.-J. Jang J.-H. Park J.-W. (2016). Modulating macrophage polarization with divalent cations in nanostructured titanium implant surfaces. *Nanotechnology* 27:085101 10.1088/0957-4484/27/8/085101 26807875

[B33] Li Y. Li J. Zhu S. Luo E. Feng G. Chen Q. (2012). Effects of strontium on proliferation and differentiation of rat bone marrow mesenchymal stem cells. *Biochem. Biophys. Res. Commun.* 418 725–730. 10.1016/j.bbrc.2012.01.088 22306818

[B34] Li Y. Li Q. Zhu S. Luo E. Li J. Feng G. (2010). The effect of strontium-substituted hydroxyapatite coating on implant fixation in ovariectomized rats. *Biomaterials* 31 9006–9014. 10.1016/j.biomaterials.2010.07.112 20800275

[B35] Lin K. Liu P. Wei L. Zou Z. Zhang W. Qian Y. (2013). Strontium substituted hydroxyapatite porous microspheres: surfactant-free hydrothermal synthesis, enhanced biological response and sustained drug release. *Chem. Eng. J.* 222 49–59. 10.1016/j.cej.2013.02.037

[B36] Liu F. Zhang X. Yu X. Xu Y. Feng T. Ren D. (2011). In vitro study in stimulating the secretion of angiogenic growth factors of strontium-doped calcium polyphosphate for bone tissue engineering. *J. Mater. Sci. Mater. Med.* 22 683–692. 10.1007/s10856-011-4247-1 21287239

[B37] Loi F. Córdova L. A. Pajarinen J. Lin T.-H. Yao Z. Goodman S. B. (2016). Inflammation, fracture and bone repair. *Bone* 86 119–130. 10.1016/j.bone.2016.02.020 26946132 PMC4833637

[B38] Mao Y. Pan M. Yang H. Lin X. Yang L. (2020). Injectable hydrogel wound dressing based on strontium ion cross-linked starch. *Front. Mater. Sci.* 14 232–241. 10.1007/s11706-020-0508-6

[B39] Marques C. F. Lemos A. Vieira S. I. Da Cruz E. Silva O. A. B. Bettencourt A. (2016). Antibiotic-loaded Sr-doped porous calcium phosphate granules as multifunctional bone grafts. *Ceram. Int.* 42 2706–2716. 10.1016/j.ceramint.2015.11.001

[B40] Marx D. Rahimnejad Yazdi A. Papini M. Towler M. (2020). A review of the latest insights into the mechanism of action of strontium in bone. *Bone Rep.* 12:100273 10.1016/j.bonr.2020.100273 PMC7210412 32395571

[B41] Meunier P. J. Roux C. Seeman E. Ortolani S. Badurski J. E. Spector T. D. (2004). The effects of strontium ranelate on the risk of vertebral fracture in women with postmenopausal osteoporosis. *New Engl. J. Med.* 350 459–468. 14749454 10.1056/NEJMoa022436

[B42] Neves N. Linhares D. Costa G. Ribeiro C. C. Barbosa M. A. (2017). In vivo and clinical application of strontium-enriched biomaterials for bone regeneration. *Bone Joint Res.* 6 366–375. 10.1302/2046-3758.66.bjr-2016-0311.r1 28600382 PMC5492369

[B43] Peng S. Zhou G. Luk K. D. K. Cheung K. M. C. Li Z. Lam W. M. (2009). Strontium promotes osteogenic differentiation of mesenchymal stem cells through the Ras/MAPK signaling pathway. *Cell. Physiol. Biochem.* 23 165–174. 10.1159/000204105 19255511

[B44] Penido M. G. M. G. Alon U. S. (2012). Phosphate homeostasis and its role in bone health. *Pediatr. Nephrol.* 27 2039–2048. 10.1007/s00467-012-2175-z 22552885 PMC3461213

[B45] Prabha R. D. Nair B. P. Ditzel N. Kjems J. Nair P. D. Kassem M. (2019). Strontium functionalized scaffold for bone tissue engineering. *Mater. Sci. Eng. C* 94 509–515. 10.1016/j.msec.2018.09.054 30423735

[B46] Reginster J. Kaufman J. Goemaere S. Devogelaer J. Benhamou C. Felsenberg D. (2012). Maintenance of antifracture efficacy over 10 years with strontium ranelate in postmenopausal osteoporosis. *Osteoporosis Int.* 23 1115–1122. 10.1007/s00198-011-1847-z 22124575 PMC3277702

[B47] Renaudin G. Laquerrière P. Filinchuk Y. Jallot E. Nedelec J. M. (2008). Structural characterization of sol–gel derived Sr-substituted calcium phosphates with anti-osteoporotic and anti-inflammatory properties. *J. Mater. Chem.* 18 3593–3600. 10.1039/b804140g

[B48] Römer P. Desaga B. Proff P. Faltermeier A. Reicheneder C. (2012). Strontium promotes cell proliferation and suppresses IL-6 expression in human PDL cells. *Ann. Anat. Anatomischer Anzeiger* 194 208–211. 10.1016/j.aanat.2011.09.008 22051238

[B49] Saberianpour S. Heidarzadeh M. Geranmayeh M. H. Hosseinkhani H. Rahbarghazi R. Nouri M. (2018). Tissue engineering strategies for the induction of angiogenesis using biomaterials. *J. Biol. Eng.* 12:36. 10.1186/s13036-018-0133-4 PMC6307144 30603044

[B50] Saidak Z. Marie P. J. (2012). Strontium signaling: molecular mechanisms and therapeutic implications in osteoporosis. *Pharmacol. Ther.* 136 216–226. 10.1016/j.pharmthera.2012.07.009 22820094

[B51] Schumacher M. Lode A. Helth A. Gelinsky M. (2013). A novel strontium(II)-modified calcium phosphate bone cement stimulates human-bone-marrow-derived mesenchymal stem cell proliferation and osteogenic differentiation in vitro. *Acta Biomater.* 9 9547–9557. 10.1016/j.actbio.2013.07.027 23917042

[B52] Sila-Asna M. Bunyaratvej A. Maeda S. Kitaguchi H. Bunyaratavej N. (2007). Osteoblast differentiation and bone formation gene expression in strontium-inducing bone marrow mesenchymal stem cell. *Kobe J. Med. Sci.* 53 25–35. 17579299

[B53] Smith L. (1963). Ceramic-plastic material as a bone substitute. *Arch. Surg.* 87 653–661. 10.1001/archsurg.1963.01310160115023 14056248

[B54] Surdacka A. Stopa J. Torlinski L. (2007). In Situ effect of strontium toothpaste on artificially decalcified human enamel. *Biol. Trace Elem. Res.* 116 147–153. 10.1007/s12011-007-9024-0 17646684

[B55] Tao Z. Zhou W. Jiang Y. Wu X. Xu Z. Yang M. (2018). Effects of strontium-modified calcium phosphate cement combined with bone morphogenetic protein-2 on osteoporotic bone defects healing in rats. *J. Biomater. Appl.* 33 3–10. 10.1177/0885328218765847 29554840

[B56] Tao Z.-S. Bai B.-L. He X.-W. Liu W. Li H. Zhou Q. (2016a). A comparative study of strontium-substituted hydroxyapatite coating on implant’s osseointegration for osteopenic rats. *Med. Biol. Eng. Comput.* 54 1959–1968. 10.1007/s11517-016-1494-9 27099156

[B57] Tao Z.-S. Zhou W.-S. He X.-W. Liu W. Bai B.-L. Zhou Q. (2016b). A comparative study of zinc, magnesium, strontium-incorporated hydroxyapatite-coated titanium implants for osseointegration of osteopenic rats. *Mater. Sci. Eng. C* 62 226–232. 10.1016/j.msec.2016.01.034 26952418

[B58] Thormann U. Ray S. Sommer U. Elkhassawna T. Rehling T. Hundgeburth M. (2013). Bone formation induced by strontium modified calcium phosphate cement in critical-size metaphyseal fracture defects in ovariectomized rats. *Biomaterials* 34 8589–8598. 10.1016/j.biomaterials.2013.07.036 23906515

[B59] Vavken J. Mameghani A. Vavken P. Schaeren S. (2016). Complications and cancer rates in spine fusion with recombinant human bone morphogenetic protein-2 (rhBMP-2). *Eur. Spine J.* 25 3979–3989. 10.1007/s00586-015-3870-9 25772092

[B60] Von Euw S. Wang Y. Laurent G. Drouet C. Babonneau F. Nassif N. (2019). Bone mineral: new insights into its chemical composition. *Sci. Rep.* 9:8456. 10.1038/s41598-019-44620-6 PMC6560110 31186433

[B61] Wang R. Wang R. Chen D. Qin G. Zhang E. (2020). Novel CoCrWNi alloys with Cu addition: microstructure, mechanical properties, corrosion properties and biocompatibility. *J. Alloys Compounds* 824:153924 10.1016/j.jallcom.2020.153924

[B62] Wang W. Yeung K. W. K. (2017). Bone grafts and biomaterials substitutes for bone defect repair: a review. *Bioactive Mater.* 2 224–247. 10.1016/j.bioactmat.2017.05.007 29744432 PMC5935655

[B63] Wang X. Wang Y. Li L. Gu Z. Xie H. Yu X. (2014). Stimulations of strontium-doped calcium polyphosphate for bone tissue engineering to protein secretion and mRNA expression of the angiogenic growth factors from endothelial cells in vitro. *Ceram. Int.* 40 6999–7005. 10.1016/j.ceramint.2013.12.027

[B64] Wornham D. P. Hajjawi M. O. Orriss I. R. Arnett T. R. (2014). Strontium potently inhibits mineralisation in bone-forming primary rat osteoblast cultures and reduces numbers of osteoclasts in mouse marrow cultures. *Osteoporosis Int.* 25 2477–2484. 10.1007/s00198-014-2791-5 25048011 PMC4176572

[B65] Wu Y. Hou J. Yin M. Wang J. Liu C. (2014). Enhanced healing of rabbit segmental radius defects with surface-coated calcium phosphate cement/bone morphogenetic protein-2 scaffolds. *Mater. Sci. Eng. C* 44 326–335. 10.1016/j.msec.2014.08.020 25280712

[B66] Xie H. Gu Z. He Y. Xu J. Xu C. Li L. (2018). Microenvironment construction of strontium–calcium-based biomaterials for bone tissue regeneration: the equilibrium effect of calcium to strontium. *J. Mater. Chem. B* 6 2332–2339. 10.1039/c8tb00306h 32254572

[B67] Xu Y. An L. Chen L. Xu H. Zeng D. Wang G. (2018). Controlled hydrothermal synthesis of strontium-substituted hydroxyapatite nanorods and their application as a drug carrier for proteins. *Adv. Powder Technol.* 29 1042–1048. 10.1016/j.apt.2018.01.008

[B68] Yan S. Feng L. Zhu Q. Yang W. Lan Y. Li D. (2018). Controlled release of BMP-2 from a heparin-conjugated strontium-substituted nanohydroxyapatite/silk fibroin scaffold for bone regeneration. *ACS Biomat. Sci. Eng.* 4, 3291–3303. 10.1021/acsbiomaterials.8b00459 33435067

[B69] Ye H. Zhu J. Deng D. Jin S. Li J. Man Y. (2019). Enhanced osteogenesis and angiogenesis by PCL/chitosan/Sr-doped calcium phosphate electrospun nanocomposite membrane for guided bone regeneration. *J. Biomater. Sci. Polym. Ed.* 30 1505–1522. 10.1080/09205063.2019.1646628 31322979

[B70] Zhao F. Lei B. Li X. Mo Y. Wang R. Chen D. (2018). Promoting in vivo early angiogenesis with sub-micrometer strontium-contained bioactive microspheres through modulating macrophage phenotypes. *Biomaterials* 178 36–47. 10.1016/j.biomaterials.2018.06.004 29908343

[B71] Zhu H. Guo D. Sun L. Li H. Hanaor D. A. H. Schmidt F. (2018). Nanostructural insights into the dissolution behavior of Sr-doped hydroxyapatite. *J. Eur. Ceram. Soc.* 38 5554–5562. 10.1016/j.jeurceramsoc.2018.07.056

